# Motor symptom asymmetry predicts non-motor outcome and quality of life following STN DBS in Parkinson's disease

**DOI:** 10.1038/s41598-022-07026-5

**Published:** 2022-02-22

**Authors:** Philippe Voruz, Jordan Pierce, Kévin Ahrweiller, Claire Haegelen, Paul Sauleau, Sophie Drapier, Dominique Drapier, Marc Vérin, Julie Péron

**Affiliations:** 1 Clinical and Experimental Neuropsychology Laboratory, Faculty of Psychology and Educational Sciences, 40 bd du Pont d’Arve, 1205 Geneva, Switzerland; 2 Neuropsychology Unit, Neurology Department, University Hospitals of Geneva, Geneva, Switzerland; 3 ‘Behavior and Basal Ganglia’ Research Unit, University of Rennes 1-Rennes University Hospital, Rennes, France; 4 Neurology Department, Pontchaillou Hospital, Rennes University Hospital, Rennes, France; 5 Neurosurgery Department, Pontchaillou Hospital, Rennes University Hospital, Rennes, France; 6 MediCIS, INSERM-University of Rennes 1, Rennes, France; 7 Physiology Department, Pontchaillou Hospital, Rennes University Hospital, Rennes, France; 8 Adult Psychiatry Department, Guillaume Régnier Hospital, Rennes, France

**Keywords:** Neurology, Neurological disorders, Psychiatric disorders

## Abstract

Risk factors for long-term non-motor symptoms and quality of life following subthalamic nucleus deep brain stimulation (STN DBS) have not yet been fully identified. In the present study, we investigated the impact of motor symptom asymmetry in Parkinson’s disease. Data were extracted for 52 patients with Parkinson’s disease (half with predominantly left-sided motor symptoms and half with predominantly right-sided ones) who underwent bilateral STN and a matched healthy control group. Performances for cognitive tests, apathy and depression symptoms, as well as quality-of-life questionnaires at 12 months post-DBS were compared with a pre-DBS baseline. Results indicated a deterioration in cognitive performance post-DBS in patients with predominantly left-sided motor symptoms. Performances of patients with predominantly right-sided motor symptoms were maintained, except for a verbal executive task. These differential effects had an impact on patients’ quality of life. The results highlight the existence of two distinct cognitive profiles of Parkinson’s disease, depending on motor symptom asymmetry. This asymmetry is a potential risk factor for non-motor adverse effects following STN DBS.

## Introduction

Although subthalamic nucleus deep brain stimulation (STN DBS) generally results in positive motor outcomes for patients with Parkinson’s disease (PD), the literature contains equivocal findings regarding its impact on cognition, with studies indicating negative effects^1,2^, although some have shown positive effects^3–5^. Studies of the neuropsychiatric effects of STN DBS^6,7^ are also far from unanimous, as some have reported a positive effect of STN DBS on neuropsychiatric symptoms^5^, while others have shown negative effects for apathy^8^, depressive symptoms and anxiety^9^, and emotion processing (for review see,^10^). As a consequence of these mixed results, current findings on quality of life in patients post-DBS are heterogeneous (for review see^6,11^).

One possible explanation for conflicting reports about the nature of STN DBS effects on cognitive performance, neuropsychiatric status, and quality-of-life measures is the heterogeneity of patients included in the studies. Recent studies in PD have focused on the impact of motor asymmetry on both cognition (for review see^12^) and social perceptual functions^13^ in pre-DBS conditions. They have highlighted different cognitive profiles for patients with PD who exhibit predominantly left-sided motor symptoms (LPD) versus those who exhibit predominantly right-sided motor symptoms (RPD), suggesting that LPD patients perform more poorly on tasks in the visuospatial modality, whereas RPD patients perform more poorly on tasks in the verbal modality or involving emotion processing^12^. A magnetic resonance imaging study also highlighted hemispheric differences in cortical degeneration in PD characterized by early left cortical atrophy, independent of motor symptom asymmetry^14^. Despite these indications of laterality effects in pre-DBS conditions, little research has been done on the non-motor impact of motor symptom asymmetry in patients post-DBS. In one recent study^15^, the authors found that LPD patients had impaired vocal emotion recognition for neutral, angry, happy, and sad voices before DBS that normalized after DBS, whereas RPD patients performed comparably to controls before DBS, but differed significantly on fear recognition after DBS. Although no significant difference was found between the RPD and LPD subgroups on any of the neuropsychological background tests in either the pre- or postoperative conditions, comparisons with healthy controls (HCs) suggested a potentially greater post-DBS deterioration in cognitive performance in LPD patients than in RPD patients. Regarding neuropsychiatric status, RPD patients scored higher on anxiety than did LPD patients in the preoperative condition, but no significant difference was found in the postoperative condition. In addition, RPD patients were more depressed than HCs in both the pre- and postoperative conditions, whereas LPD patients showed no depressive symptoms at any point. Finally, no effect was reported for apathy. These results suggest that neuropsychiatric profiles differ according to motor symptom asymmetry, with greater vulnerability of RPD patients but also a beneficial effect of DBS on anxiety in this subgroup. Given STN functional anatomy, these results suggest that DBS has a dissociative effect in the cognitive and affective domains, depending on motor symptom lateralization. Based on the integrative model of STN function put forward by Péron, et al.^10^, the study by Voruz, et al.^15^ supports the notion that although STN DBS typically restores motor functions, the surgery/stimulation can unintentionally impair non-motor functions. In this study, RPD patients (with inferred left cerebral pathology) did not exhibit any emotional impairment prior to surgery (probably owing to right hemispheric specialization of the basal ganglia for vocal emotion processing^16^), but developed emotional impairment in the wake of DBS. In contrast, LPD patients did display vocal emotional impairment prior to surgery (in line with the study by Stirnimann, et al.^16^), but this deficit normalized post-DBS. A recent study that used intracranial recordings corroborated this notion by showing that STN oscillations during emotion processing vary according to PD motor asymmetry^17^. From a clinical perspective, this could be crucial for patient management, as it points to a dissociative effect across the cognitive, neuropsychiatric, and motor domains post-DBS according to motor symptom asymmetry. To our knowledge, however, this has not yet been studied in a larger cohort in a pre- versus post-DBS condition, as in Voruz, et al.^15^ study.

In this context, our primary objective in the present study was to investigate changes in cognitive symptoms 12 months after DBS surgery in a cohort of 52 patients in comparison with the results of a baseline assessment conducted 3 months pre-DBS and the performances of an HC group. Patients were classified according to the lateralization of their motor symptoms (LPD vs. RPD). Our secondary objective was to measure the influence of motor symptom asymmetry and DBS on neuropsychiatric characteristics and physical and mental quality of life.

From overall findings in the literature, we made several predictions about the expected effects of motor symptom asymmetry on cognition, apathy and depression symptoms, and quality of life following DBS. First, on the basis of Voruz, et al.^15^ results in the neuropsychological domain, we expected to observe poorer performance on cognitive function tests post-DBS, but only for the LPD subgroup, with performance levels unchanged in the RPD subgroup. Second, we predicted that RPD patients would exhibit greater vulnerability to apathy and depression symptoms than would LPD patients and that DBS would have a beneficial effect on these symptoms in the RPD subgroup^15^. Third, from the results of Drapier, et al.^7^, we predicted that DBS would bring about an improvement in quality of life and that this improvement would be driven by the physical quality-of-life subscore. This improvement would be significant only in RPD patients, driven by a significant improvement in their motor symptoms, as observed by Voruz, et al.^15^.

## Methods

### Participants

As described elsewhere^18^, we extracted data from the Rennes Hospital database for 52 patients with PD (26 RPD and 26 LPD patients) who had undergone bilateral STN DBS between 2004 and 2015, matched on age, sex, handedness, socio-education level, and disease duration. All patients met the criteria of the Parkinson’s UK Brain Bank for idiopathic PD^19^. Selection criteria for STN DBS followed international recommendations published in 1999^20^ that include disabling levodopa (DOPA)-induced symptoms refractory to medical treatment (motor fluctuations and dyskinesias), disease > 5 years, and age < 70 years. All patients were implanted bilaterally in the STN with quadripolar DBS electrodes (monopolar stimulation). The locations of the electrodes were determined by using stereotactic coordinates derived from a three-dimensional computed tomography scan (coordinate of active electrode in Table 1). General information regarding STN DBS parameters are available in Table 1. Motor symptoms were assessed by using Part III of the Unified Parkinson’s Disease Rating Scale (UPDRS)^21^, the Hoehn and Yahr Scale (H&Y;^22^), the Schwab and England Scale (S&E;^23^), and the Activities of Daily Living scale^24^ in on- and off-DOPA conditions. The total levodopa-equivalent daily dose (LEDD) was calculated in accordance with Lozano, et al.^25^. The asymmetry of motor symptoms prior to DBS was retrospectively calculated on the lateralized items (items 20–26) of the UPDRS III^21^. Patients with a negative score were considered to be LPD patients and those with a positive score were considered to be RPD patients. None of the participants exhibited cognitive decline as measured with the Mattis Dementia Rating Scale^26^ (MDRS > 130), major executive decline, or DOPA-resistant axial motor signs.

**Table 1:** Sociodemographic data of the LPD (*n* = 26) and RPD (*n* = 26) subgroups of patients with PD and of the HC group (*n* = 25), and stimulation parameters and location of the active electrode for the patient subgroups.

	RPD Mean ± *SD*	LPD Mean ± *SD*	HC Mean ± *SD*
**Sociodemographic data**			
Age	57.19 ± 7.41	57.31 ± 6.80	54.48 ± 8.50
Sex (F/M)	14/12	11/15	12/13
Handedness (R/L)	24/2	24/2	24/1
Socio-education level (levels 1–4)^b^	3.19 ± 0.98	3.23 ± 1.07	3.12 ± 0.93
Disease duration (years)	11.73 ± 3.86	11.27 ± 4.22	NA
**STN DBS electrode location**			
Right location X	14.65 ± 2.57	13.24 ± 2.04	NA
Right location Y	− 16.54 ± 1.97	− 16.18 ± 1.65	NA
Right location Z	− 1.50 ± 2.42	− 1.67 ± 3.41	NA
Left location X	− 14.13 ± 1.59	− 14.48 ± 1.39	NA
Left location Y	− 16.84 ± 1.91	− 16.43 ± 1.68	NA
Left location Z	− 1.41 ± 1.80	− 1.64 ± 2.19	NA
**DBS stimulation parameters**			
DBS—Right voltage (V)	2.11 ± 0.65	2.49 ± 0.68^a^	NA
DBS—Right pulse width (µs)	61.15 ± 5.88	62.31 ± 8.15	NA
DBS—Right frequency (Hz)	132.31 ± 10.79	133.27 ± 6.92	NA
DBS—Left pulse width (V)	2.42 ± 0.64	2.03 ± 0.69	NA
DBS—Left time (µs)	61.15 ± 5.88	61.15 ± 5.88	NA
DBS—Left frequency (Hz)	132.89 ± 11.06	132.12 ± 6.03	NA

*F* female, *HC* healthy control, *Hz* hertz, *L* left, *LPD* patients with Parkinson’s disease (PD) who exhibit predominantly left-sided motor symptoms, *M* male, *NA* not applicable, *R* right, *RPD* patients with PD who exhibit predominantly right-sided motor symptoms, *SD* standard deviation; µs: microsecond; *V* volt.

^a^*p* < 0.05 compared with RPD subgroup (Mann–Whitney *U* test).

^b^The socio-cultural level was determined by the number of years of study (level 1: compulsory school and below; level 2: vocational training; level 3: baccalaureate; level 4: higher studies).

**Table 2:** Neuropsychological and cognitive performances of the LPD (*n* = 26) and RPD (*n* = 26) subgroups of patients with PD before (preoperative condition, baseline) and after (postoperative condition, M + 12) STN DBS and of the HC group (*n* = 25).

	RPD		LPD		HC
	Preoperative Mean ± *SD*	12 months postoperative Mean ± *SD*	Preoperative Mean ± *SD*	12 months postoperative Mean ± *SD*	Mean ± *SD*
**Global cognitive efficiency**					
MDRS (/144)^a26^	141.19 ± 2.53	140.73 ± 2.47	139.88 ± 3.00	139.81 ± 3.07	141.68 ± 1.70
MDRS—Attention^a^	36.53 ± 0.65	36.38 ± 0.64	36.58 ± 0.81	36.58 ± 0.70	–
MDRS—Initiation^a^	36.53 ± 1.39	36.19 ± 1.90	35.65 ± 2.21	35.46 ± 2.47	–
MDRS—Construction^a^	6.00 ± 0.00	5.96 ± 0.20	6.00 ± 0.00	6.00 ± 0.00	-
MDRS—Conceptualization^a^	38.23 ± 1.03	38.12 ± 1.14	38.08 ± 0.98	37.81 ± 1.23	–
MDRS—Memory^a^	23.88 ± 1.34	24.08 ± 1.09	23.62 ± 1.63	23.96 ± 1.68	–
**Executive functions**					
Stroop Test—Interference^a^	1.54 ± 7.32	2.73 ± 5.40	1.82 ± 6.52	3.62 ± 6.66	3.09 ± 6.83
TMT B-A (seconds)^b^	63.08 ± 46.71	52.77 ± 41.68	64.15 ± 61.67	74.88 ± 72.86*	42.56 ± 23.87
Categorical verbal fluency (2')^a^	29.54 ± 11.87	26.73 ± 9.14**	27.35 ± 10.71**	27.23 ± 9.24*	32.85 ± 7.45
Phonemic verbal fluency (2')^a^	22.50 ± 8.24	21.42 ± 7.76	19.08 ± 6.66	18.62 ± 5.64	20.96 ± 6.29
MCST—Number of categories (/6)^a^	5.83 ± 0.93	5.78 ± 0.50	5.54 ± 0.86	5.15 ± 1.56**	5.96 ± 0.20
MCST—Number of errors^b^	4.88 ± 4.63	4.50 ± 4.16	5 ± 5.52	7.17 ± 7.16	2.80 ± 2.10
MCST—Number of perseverations^b^	1.27 ± 1.66	1.04 ± 1.64	1.54 ± 2.17	2.58 ± 3.21	0.56 ± 0.77

Differential effects between the two conditions are reported.

*HC* healthy control, *LPD* patients with Parkinson’s disease (PD) exhibiting predominantly left-sided motor symptoms, *MCST* modified Wisconsin card sorting test, *MDRS* mattis dementia rating scale, *RPD* patients with PD exhibiting predominantly right-sided motor symptoms, *SD* standard deviation, *STN DBS* subthalamic nucleus deep brain stimulation, *TMT* trail making test.

*p < 0.05 compared with HC group.

**Significant after FDR correction compared with HC group.

^a^The higher the score, the better the performance.

^b^The lower the score, the better the performance.

Patients completed a clinical assessment (motor, cognitive, apathy, depression, and quality-of-life batteries) 3 months before the operation (on DOPA medication) and 12 months after it (on DOPA medication and on stimulation). Patient subgroups were compared with 25 HCs matched for age, sex, and sociocultural level (see Table 1), who had no history of neurological disease, head injury, or alcohol abuse and who displayed no signs of dementia.

The study was approved by the ethics committee of Rennes University Hospital and conducted in accordance with the Declaration of Helsinki. All patients gave their written informed consent before taking part.

### Cognitive, apathy, depression, and quality-of-life assessments

Cognitive, apathy, depression, and quality-of-life assessments were administered to all patients included in the Rennes University Hospital cohort.

The cognitive battery included the MDRS^26^ and a series of tests that assessed frontal executive functions: the Modified Wisconsin Card Sorting Test (MCST,^27^, the Trail Making Test (TMT,^28^, the Categorical and Literal Fluency Test (2’)^29^, and the Stroop test^30^.

The assessment for depression and apathy symptoms included the Montgomery-Åsberg Depression Rating Scale (MADRS,^31^), chosen because of the predominance of psychological items over somatic items, thus limiting interference with PD symptoms, and the Apathy Evaluation Scale (AES,^32^), which yields a general apathy score, as well as subscores for emotional (AES-E), social (AES-S), and other (AES-O) apathy.

Finally, quality of life was assessed by using the Short Form 36-item Health Survey (SF-36,^33^, which yields separate mental and physical quality-of-life scores.

### Statistical analysis

To compare the effects of DBS at different time points among LPD and RPD patients, as well as the differences between patients and HC, we ran one-tailed independent-sample *t* tests for intergroup comparisons and one-tailed dependent-sample *t* tests for intragroup comparisons on the normally distributed variables. For those variables with non-normal distributions (i.e., DBS parameters, MDRS, MCST), we performed a Kruskal–Wallis nonparametric one-way analysis of variance on ranks for intergroup comparisons between more than two groups (LPD patients 3 months pre-DBS; LPD patients 12 months post-DBS; RPD patients 3 months pre-DBS; RPD patients 12 months post-DBS; and HC group) and Mann–Whitney *U* tests if there was a significant group effect. For the nonparametric intragroup comparisons, we performed Wilcoxon matched pairs tests. All significant results (*p* < 0.05) are indicated in the tables according to the conditions being compared. Because we performed planned comparisons on the basis of operational hypotheses on a few sensible features and we report the results of all analyses and given the descriptive and advisory nature of this study, corrections for multiple comparisons were not performed. Nevertheless, to avoid an effect of inter-collinearity in a second step, we performed a false discovery rate (FDR) correction at each time point (pre- and post-DBS condition) and for each domain of results (motor, cognition, apathy/depression, and quality of life).

To quantify the relationships between the cognitive variables, sociodemographic factors, stimulation parameters, motor symptoms, neuropsychiatric variables, and quality-of-life data, we ran linear multiple regression forward stepwise analyses on ∆ scores (12-month post-DBS score—3-month pre-DBS score) for all variables. To reduce the likelihood of Type I errors, only variables that were found to differ significantly in intra- or intergroup comparisons were included in the models.

Analyses were performed with Statistica version 13.5.0.

## Results

### Stimulation parameters and electrode location

Interestingly, we observed a significant difference in voltage parameters between RPD and LPD subgroups (Table 1). LPD patients had a higher voltage than RPD patients did in the right STN (*z* = − 2.21, *p* = 0.02), and RPD patients tended to have a higher voltage than LPD patients did in the left STN (*z* = 1.73, *p* = 0.08). No other significant difference was found in stimulation parameters between LPD and RPD subgroups. No significant difference was found for electrode location, although it should be noted that data concerning electrode contact coordinates were missing for 21 patients.

### DOPA therapy and motor effects of STN DBS (Tables 1 and 2)

A significant decrease in LEDD was observed in the postoperative condition in comparison with the preoperative condition within the two subgroups, whereas no significant difference was observed in intergroup comparisons.

There was a significant intragroup difference for RPD and LPD patients who were on medication and on stimulation regarding the UPDRS III score between the pre-DBS and the 12-month post-DBS conditions (Supplementary Table 1). Interestingly, intergroup analyses revealed a significant difference between the two subgroups on the S&E score in the off-DOPA condition at 12 months post-DBS (*t* = 2.57, *p* = 0.013, significant after FDR correction), with a higher score for LPD patients (Supplementary Table 2. All other results were nonsignificant.

### Effects of STN DBS on cognition, apathy, depression, and quality of life

#### Cognition (Table 2 and Fig. 1)

In the pre-DBS condition, results revealed a significant difference between the LPD subgroup and the HCs on categorical verbal fluency (*t* = 2.04, *p* = 0.047).

In the post-DBS condition, results revealed no significant difference in cognitive performance between the RPD subgroup and the HC group at 12 months, except for categorical verbal fluency at 12 months post-DBS (*t* = 2.51, *p* = 0.015). However, there was a negative effect of DBS on the cognitive performance of the LPD subgroup, compared with the HC group, at 12 months. More specifically, this effect was observed for TMT B-A performance at 12 months post-DBS (*t* = − 2.11, *p* = 0.040) and MCST—Number of categories 12 months post-DBS (*z* = 2.26, *p* = 0.024).

After FDR correction, only the significant difference between RPD and HC for categorical verbal fluency at 12 months post-DBS and between LPD and HC for MCST—Number of categories at 12 months post-DBS survived. All other results were nonsignificant after FDR correction.

#### Apathy and depression (Supplementary Table 3)

*Apathy*. Intergroup analyses revealed a significant difference in the pre-DBS condition between RPD and LPD patients on the AES-O (*t* =− 2.12, *p* = 0.039) and AES-E (*t* = − 2.41, *p* = 0.020) subscales, suggesting greater other and emotional apathy in RPD patients (Supplementary Table 3, but only the AES-E result survived to FDR correction. Interestingly, no significant difference was observed between these two groups at 12 months post-DBS on the AES-O (*t* = − 1.18, *p* = 0.244) and AES-E (*t* =− 2.02, *p* = 0.052) subscales.

*Depression*. Analysis also revealed a significant difference in the pre-DBS condition between RPD and LPD subgroups and the HC group on the depression scale. Both patient subgroups had higher ratings than the HC group in the preoperative condition (LPD vs. HC: z = − 2.97, *p* = 0.003; RPD vs. HC: *z* = − 2.95, *p* = 0.003) and in the 12-month postoperative condition. All results survived to FDR correction (Supplementary Table 3).

All other results were nonsignificant.

#### Quality of life (Supplementary Table 4)

The intragroup analyses of scores on the two quality-of-life questionnaires indicated significant improvements in the SF-36 total score at 12 months and mainly on the subscales for the RPD subgroup (Supplementary Table 4). The SF-36 total (*t* = − 2.26, *p* = 0.033), global health (*t* = − 3.18, *p* = 0.004), physical role (*t* = − 2.30, *p* = 0.031), physical score (*t* = − 2.90, *p* = 0.008), and social function (*t* = − 3.34, *p* = 0.003) scores improved significantly at 12 months for RPD patients. After FDR correction, for the RPD subgroup, global health, physical score, and social function were still significant, whereas a significant improvement in LPD patients was reported for only the global health (*t* = − 2.40, *p* = 0.025) and physical pain (*t* = − 2.52, *p* = 0.019) scores and both results for LPD survived to FDR correction. All other results for SF-36 were nonsignificant.

### Relationships among variables that distinguished between LPD and RPD patients and secondary measures

Significant differences were observed for the following variables of interest: on-DOPA on-stimulation UPDRS III, off-DOPA S&E, TMT B-A, categorical verbal fluency, phonemic verbal fluency, AES-E, AES-O, and SF-36 global health scores. Regression models were fitted for each variable to explore which additional measures best explained the observed group differences.

*For the on-DOPA on-stimulation UPDRS III score*, the best fit was achieved by using the SF-36 physical role score (*R*^2^ = 0.12, *p* = 0.043).

*For the off-DOPA S&E score,* the best fit was achieved by using the SF-36 physical function (*R*^2^ = 0.30, *p* = 0.002), off-DOPA H&Y (*R*^2^ = 0.10, *p* = 0.041), Stroop interference (*R*^2^ = 0.12, *p* = 0.016), AES-O (*R*^2^ = 0.08, *p* = 0.037), SF-36 physical function (*R*^2^ = 0.08, *p* = 0.022), AES-B (*R*^2^ = 0.05, *p* = 0.043), and MDRS total (*R*^2^ = 0.05, *p* = 0.031) scores.

*For the TMT B-A score*, the best fit was achieved by using the off-DOPA off-stimulation UPDRS III (*R*^2^ = 0.13, *p* = 0.037), off-DOPA H&Y (*R*^2^ = 0.10, *p* = 0.035), and SF-36 physical pain (*R*^2^ = 0.04, *p* = 0.018) scores.

*For categorical verbal fluency*, the best fit was achieved by using the LEDD (*R*^2^ = 0.14, *p* = 0.041) and SF-36 vitality (*R*^2^ = 0.18, *p* = 0.008) scores.

*For phonemic verbal fluency*, the best fit was achieved by using the SF-36 vitality (*R*^2^ = 0.28, *p* = 0.003), SF-36 physical (*R*^2^ = 0.15, *p* = 0.009), Stroop interference (*R*^2^ = 0.09, *p* = 0.028), SF-36 emotional role (*R*^2^ = 0.09, *p* = 0.016), and AES-E (*R*^2^ = 0.06, *p* = 0.024) scores.

*For the AES-E score*, the best fit was achieved by using the AES-B (*R*^2^ = 0.57, *p* < 0.001), SF-36 mental health (*R*^2^ = 0.08, *p* = 0.018), AES-Total (*R*^2^ = 0.06, *p* = 0.001), and SF-36 mental (*R*^2^ = 0.01, *p* = 0.040) scores.

*For the AES-O score*, the best fit was achieved by using the AES-Total (*R*^2^ = 0.52, *p* < 0.001), off-DOPA on-stimulation UPDRS III (*R*^2^ = 0.08, *p* = 0.026), and SF-36 physical (*R*^2^ = 0.10, *p* = 0.035) scores.

*For the SF36 global health score*, the best fit was achieved by using the off-DOPA H&Y (*R*^2^ = 0.19, *p* = 0.015), on-DOPA on-stimulation UPDRS III (*R*^2^ = 0.12, *p* = 0.035), AES-E (*R*^2^ = 0.10, *p* = 0.021), and TMT (*R*^2^ = 0.10, *p* = 0.003) scores.

## Discussion

The aim of the present study was to investigate the cognitive, neuropsychiatric (apathy and depression), and quality-of-life profiles of patients with PD 12 months after STN DBS as a function of motor symptom asymmetry. According to recent research^15^, motor symptom asymmetry could be a meaningful predictor of non-motor performance following surgery. The present results seem to indicate that, at 12 months, DBS does not have a detrimental effect on cognitive measures for RPD patients (with inferred left cerebral pathology), whereas we did observe a harmful effect for LPD patients compared with HCs. In addition, whereas apathy seemed to be reduced for RPD patients, there were no changes in the severity of neuropsychiatric symptoms (i.e., apathy and depression scores) for LPD patients. As a consequence, DBS primarily had a beneficial effect on quality of life for RPD patients. These findings indicate that PD motor symptom asymmetry is a risk factor in the non-motor impact of STN DBS.

Before taking the interpretation any further, several main limitations need to be considered. First, the retrospective nature of the study, as well as the small size of the sample, represent limitations to the inferences that can be made from the present results. Second, the contacts used for chronic stimulation, according to the coordinates X, Y, Z, which were calculated with respect to the inter-commissural (anterior commissure-posterior commissure) line, did not allow precise localization of the stimulation contacts within the STN. The unavailability of contact coordinates for 21 subjects did not allow specific speculation about the direct effects of stimulation as a function of laterality of motor symptoms, and so these effects remain to be explored in future studies. Third, in our patient groups, we had two left-handed patients in each group. Although our groups were counterbalanced, the presence of patients with reverse brain asymmetry may have influenced the results. Fourth, one can argue that dopaminergic treatment may underlie the post-DBS effects, notably on apathy found in RPD patients. That being said, we failed to observe a significant difference between RPD and LPD patients for LEDD in both the pre- and postoperative conditions, or to find a significant correlation between LEDD and AES in the multiple regression analyses. Fifth and finally, we chose to discuss all our results for multiple comparisons, despite the fact that the majority of our results were still significant after FDR correction. We realize that the significance level of 0.05 was not maintained for all hypotheses combined. Nevertheless, our choice to discuss all of our results can be explained by the descriptive and advisory nature of this study, which would have excluded relevant data for the interpretation of the results, as well as by the fact that we had previously chosen to analyze only a limited number of features related to our hypotheses in order to present all the results and that we did not carry out a post hoc analysis afterwards.

Analysis of cognitive measures revealed differential effects of DBS as a function of motor symptom asymmetry. As predicted, cognitive performances were mostly preserved in RPD patients compared with those in HCs at 12 months post-DBS, the exception being scores on the executive verbal task, where performance worsened post-DBS. In contrast, at 12 months, the performances of LPD patients worsened on nonverbal executive functions compared with those in HCs. This pattern of results corroborates the post-DBS results reported by Hershey, et al.^34^, as well as those observed by Voruz, et al.^35^. A first interpretation of these results may be that they represent an overload effect of the DBS in the cerebral hemisphere ipsilateral to motor symptoms. Indeed, as postulated by Péron, et al.^36^, DBS would induce a desynchronizing effect on this ipsilateral associative loop through possible interconnectivity between the motor and associative areas of the STN^37^, or by a direct effect of the DBS on the associative loop^38^, while restoring the synchronization of the contralateral loops. That said, how can we explain that this effect would be more prominent for LPD than for RPD patients? A more critical and complex role of the left STN may be suspected, despite interhemispheric interaction between the large-scale networks involving the two STNs^17^. In the case of LPD patients, as suggested by our results, dynamics and connectivity between the basal ganglia and other cortical regions involving fine executive processing would be altered. The LPD subgroup’s poorer performance on the TMT B-A (not significant after FDR correction), the number of categories on the MCST (still significant after FDR correction), and verbal fluency (not significant after FDR correction) could be explained by an alteration of processes of connectivity involving the prefrontal, frontal, and basal ganglia regions of the left hemisphere^39–41^. A similar phenomenon could be hypothesized for the RPD subgroup, with a potential overload effect of the DBS on the right STN, but because tasks that investigate visuo-spatial performance are lacking, these phenomena could not be observed during our study. In the future, it would be interesting to perform wider neuropsychological testing (including exhaustive memory and visuo-spatial tests) post-DBS. Finally, in the very long term, we predict that LPD patients would be able to compensate for the detrimental effect of DBS and would deteriorate less cognitively. RPD patients, on the other hand, would remain stable in the first month post-DBS (except for visuo-spatial performances for the reasons evoked earlier), but are expected to exhibit major cognitive impairment in the very long term because of the well-known vulnerability of the left hemisphere to neurodegeneration, which was illustrated in our results by the worsening of verbal executive tasks in these patients^42–44^.

Analysis revealed no significant differences between the PD subgroups for depression symptoms. However, there were significant differences between LPD and RPD patients on the AES-E and AES-O subscores (only AES-E score survived to FDR correction). At the pre-DBS assessment, RPD patients scored higher than LPD patients on these two subscales, indicating greater apathy in RPD than in LPD patients. Interestingly, at 12 months post-DBS, LPD and RPD patients no longer differed significantly on the AES-O and AES-E subscales. Although we failed to observe intragroup effects, intergroup effects suggest a differential modification in post-DBS apathy as a function of motor asymmetry. These findings may suggest greater vulnerability of RPD patients for developing apathy symptoms in the natural evolution of the disease and that DBS has the potential to restore mood disorders in this patient subtype. Previous results that showed an increase in apathy may have been biased by the presence of unequal proportions of LPD and RPD patients^45^. Future empirical and meta-analytic studies are needed, considering the motor symptom asymmetry.

Finally, in line with our predictions, results for quality of life also differed according to motor symptom asymmetry. Notably, RPD patients exhibited post-DBS improvement in their quality of life across all domains (overall, global health, social function, physical role, and physical function scores) at 12 months post-DBS, whereas LPD patients did not display an improvement in the overall and physical scores or in the social function score. These results are in line with the literature suggesting an improvement in quality of life post-DBS in whole PD studies^6^; in addition, they demonstrate a weaker effect in LPD patients. These results may suggest that the improvement of quality of life observed in whole PD groups may have been driven by the RPD subgroup of patients, but this issue remains to be explored in future studies. Moreover, as expected, motor scores significantly predicted improvement in global health but, interestingly, the apathy (AES-E) and cognitive (TMT) scores were also significant predictors and may explain why quality of life is significantly less increased for the LPD subgroup, in whom cognitive performances decrease in postoperative conditions.

## Conclusion

The current findings support the existence of two profiles of PD based on motor symptom asymmetry at disease onset that differ in terms of the impact of STN DBS on patients’ cognitive performance, apathy and depression symptoms, and mental and physical quality of life. It would therefore be useful to include asymmetry as a variable in future studies in order to better characterize these profiles and the effect of DBS on each, as well as to build better predictions of disease progression. Our results open a new avenue for research in the field of personalized medicine. An individualized approach could increase the efficacy of PD treatments, as well as prompt early recourse to cognitive-behavioral therapy in certain populations in order to limit cognitive decline.

**Figure 1:**
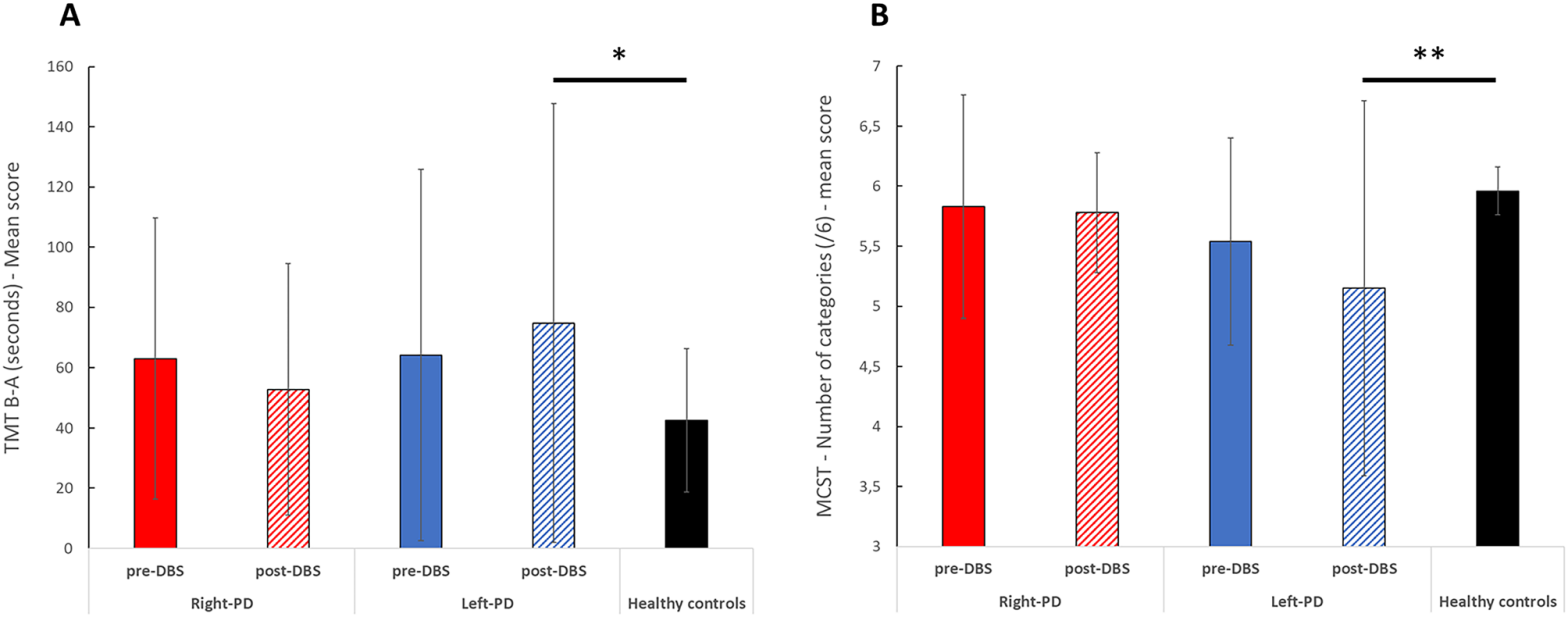
Neuropsychological and cognitive performances of the LPD and RPD subgroups of patients with PD before (preoperative condition, baseline) and after (postoperative condition, M + 12) STN DBS and of the HC group. (**A**) For mental flexibility (TMT B-A score), LPD performed significantly worse than healthy controls in the post-DBS condition, whereas no significant differences were observed in the pre-DBS condition, as well as between RPD and healthy controls for the pre- and post-DBS condition. (**B**) For MCST—Number of categories, LPD performed significantly worse than healthy controls in the post-DBS condition, whereas no significant differences were observed in the pre-DBS condition, as well as between RPD and healthy controls for the pre- and post-DBS condition. *DBS* deep brain stimulation, *LPD*: predominantly left-motor symptoms, *MCST* modified Wisconsin card sorting test, *RPD* predominantly right-motor symptoms, *TMT* trail making test. ^*^p < .050. ^**^Significant after FDR correction.

## Supplementary Information

Supplementary Information.

## Abbreviations

AES: Apathy evaluation scale
DBS: Deep brain stimulation
DOPA: Levodopa
HC: Healthy control
H and Y: Hoehn and Yahr scale
LEDD: Levodopa-equivalent daily dose
LPD: Patients with Parkinson’s disease exhibiting predominantly left-sided motor symptoms
MCST: Modified Wisconsin card sorting test
MDRS: Mattis dementia rating scale
PD: Parkinson’s disease
RPD: Patients with Parkinson’s disease exhibiting predominantly right-sided motor symptoms
S and E: Schwab and England scale
SF-36: Short form 36-item health survey
STN: Subthalamic nucleus
TMT: Trail making test
UPDRS: Unified Parkinson’s disease rating scale
