# Supplementary material for: Motor symptom asymmetry predicts non-motor outcome and quality of life following STN DBS in Parkinson's disease
Source: Sci Rep. 2022 Feb 22;12:3007. doi: 10.1038/s41598-022-07026-5 (PMC8863787; doi:10.1038/s41598-022-07026-5)
Supplement: Supplementary file 1 — Supplementary Information. [file 41598_2022_7026_MOESM1_ESM.docx]

**Supplementary Table 1. On-DOPA motor scores before (preoperative condition, baseline) and after (postoperative condition) STN DBS in patients with PD (*N* = 52), distinguishing between LPD and RPD subgroups**.

|  | **RPD on-DOPA** | | **LPD on-DOPA** | |
| --- | --- | --- | --- | --- |
|  | **Preoperative**  **Mean ± *SD*** | **12 months postoperative**  **Mean ± *SD*** | **Preoperative**  **Mean ± *SD*** | **12 months postoperative**  **Mean ± *SD*** |
| **UPDRS III** off-stimulation | 8.70 ± 5.02 | 12.14 ± 7.67^“”^ | 9.37 ± 6.78 | 13.26 ± 7.56^“”^ |
| **UPDRS III** on-stimulation | 8.70 ± 5.02 | 7.75 ± 6.28 | 9.37 ± 6.78 | 6.63 ± 4.91 |
| **S&E (%)** | 86.80 ± 6.27 | 91.54 ± 8.34^“”^ | 89.23 ± 11.97 | 93.60 ± 8.60 |
| **H&Y** | 0.95 ± 0.74 | 0.57 ± 0.74 | 0.81 ± 0.90 | 0.60 ± 0.88 |
| **LEDD** | 1211.20 ± 586.40 | 741.44 ± 443.43^“”^ | 1291.02 ± 651.91 | 753.69 ± 436.19^“^ |

Differential effects between the two conditions are reported.

^““^ Significant after FDR correction compared with preoperative condition.

**Legend:** DOPA: levodopa; STN DBS: subthalamic nucleus deep-brain stimulation; PD, Parkinson’s disease; LPD: patients with PD who exhibit predominantly left-sided motor symptoms; RPD: patients with PD who exhibit predominantly right-sided motor symptoms; *SD*: standard deviation; UPDRS: Unified Parkinson’s Disease Rating Scale; S&E: Schwab & England scale; H&Y: Hoehn & Yahr scale; LEDD: levodopa-equivalent daily dose.

**Supplementary Table 2. Off-DOPA motor scores before (preoperative condition, baseline) and after (postoperative condition) STN DBS in patients with PD (*N* = 52), distinguishing between LPD and RPD subgroups**.

|  | **RPD Off-DOPA** | | **LPD Off-DOPA** | |
| --- | --- | --- | --- | --- |
|  | **Pre-op**  **Mean ± *SD*** | **12 months post-op**  **Mean ± *SD*** | **Pre-op**  **Mean ± *SD*** | **12 months post-op**  **Mean ± *SD*** |
| **UPDRS III** off-stimulation | 32.60 ± 11.15 | 34.56 ± 15.78 | 32.50 ± 11.55 | 34.16 ± 13.86 |
| **UPDRS III** on-stimulation | 32.60 ± 11.15 | 17.60 ± 10.71^“”^ | 32.50 ± 11.55 | 17.56 ± 9.24^“”^ |
| **S&E (%)** | 60.40 ± 20.51 | 72.31 ± 19.25^“”^ | 69.62 ± 19.69 | 83.20 ± 9.00^@@“”^ |
| **H&Y** | 2.63 ± 1.09 | 2.00 ± 0.97 | 2.24 ± 0.90 | 1.90 ± 0.81 |

Differential effects between the two conditions are reported (*t* test for dependent groups).

^@@^ Significant after FDR correction compared with RPD subgroup.

^““^ Significant after FDR correction compared with preoperative condition.

**Legend:** DOPA: levodopa; STN DBS: subthalamic nucleus deep-brain stimulation; PD, Parkinson’s disease; LPD: patients with PD who exhibit predominantly left-sided motor symptoms; RPD: patients with PD who exhibit predominantly right-sided motor symptoms; *SD*: standard deviation; UPDRS: Unified Parkinson’s Disease Rating Scale; S&E: Schwab & England scale; H&Y: Hoehn & Yahr scale.

**Supplementary Table 3. Depressive and apathy scores of LPD (*n* = 26) and RPD (*n* = 26) subgroups of patients with PD before (preoperative condition, baseline; *n* = 52) and after (postoperative condition; *n* = 52) STN DBS and of the HC group (*n* = 25).**

|  | | **RPD** | | **LPD** | | **HC** |
| --- | --- | --- | --- | --- | --- | --- |
|  | | **Preoperative**  **Mean ± *SD*** | **12 months postoperative**  **Mean ± *SD*** | **Preoperative**  **Mean ± *SD*** | **12 months postoperative**  **Mean ± *SD*** | **Mean ± *SD*** |
| **Depression** | **MADRS**^-^ | 4.09 ± 3.27^**^ | 6.25 ± 5.62^**^ | 5.46 ± 5.72^**^ | 4.44 ± 4.31^**^ | 1.29 ± 2.08 |
| **Apathy** | **AES Total**^-^ | 32.62 ± 5.99 | 33.74 ± 9.33 | 29.36 ± 8.26 | 31.65 ± 6.53 | - |
|  | AES – C^-^ | 14.38 ± 2.59 | 14.95 ± 4.20 | 13.12 ± 3.77 | 14.88 ± 2.93 | - |
|  | AES – B^-^ | 8.19 ± 1.90 | 8.37 ± 2.81 | 7.88 ± 2.28 | 7.76 ± 1.56 | - |
|  | AES – E^-^ | 4.50 ± 0.86 | 4.47 ± 1.02 | 3.76 ± 1.30^@@^ | 3.76 ± 1.09 | - |
|  | AES – O^-^ | 5.54 ± 1.45 | 5.95 ± 2.01 | 4.60 ± 1.71^@^ | 5.24 ± 1.52 | - |

Differential effects between the two subgroups are reported.

^**^ Significant after FDR correction compared with HC group.

^@^ *p* < 0.05 compared with RPD subgroup.

^@@^ Significant after FDR correction compared with RPD subgroup.

**Legend:** LPD: patients with Parkinson’s disease (PD) who exhibit predominantly left-sided motor symptoms; RPD: patients with PD who exhibit predominantly right-sided motor symptoms; STN DBS: subthalamic nucleus deep-brain stimulation; HC: healthy control; *SD*: standard deviation; MADRS: Montgomery‑Åsberg Depression Rating Scale; AES: Apathy Evaluation Scale.

^-^ The lower the score, the better the mood.

**Supplementary Table 4. Quality-of-life scores of LPD (*n* = 26) and RPD (*n* = 26) subgroups of patients with PD before (preoperative condition, baseline) and after (postoperative condition) STN DBS and of the HC group (*n* = 25).**

|  | | **RPD** | | **LPD** | |
| --- | --- | --- | --- | --- | --- |
|  | | **Preoperative**  **Mean ± *SD*** | **12 months postoperative**  **Mean ± *SD*** | **Preoperative**  **Mean ± *SD*** | **12 months postoperative**  **Mean ± *SD*** |
| **Quality of life** | **SF-36 Total**^+^ | 49.22 ± 13.24 | 59.49 ± 21.33^“^ | 53.48 ± 19.04 | 59.83 ± 20.23 |
|  | SF-36 – Global health^+^ | 40.38 ± 18.05 | 48.80 ± 17.58^“”^ | 49.42 ± 18.51 | 55.60 ± 17.76^“”^ |
|  | SF-36 – Physical function^+^ | 65.38 ± 19.02 | 72.20 ± 20.87 | 61.92 ± 26.23 | 63.96 ± 27.39 |
|  | SF-36 – Physical role^+^ | 23.08 ± 33.85 | 48 ± 46.73^“^ | 34.62 ± 38.13 | 44.00 ± 43.47 |
|  | SF-36 – Emotional role^+^ | 57.69 ± 43.78 | 64 ± 47.06 | 58.97 ± 43.52 | 66.67 ± 44.00 |
|  | SF-36 – Social function^+^ | 52.00 ± 19.33 | 68.50 ± 23.42^“”^ | 59.13 ± 23.06 | 62.00 ± 21.19 |
|  | SF-36 – Physical pain^+^ | 53.00 ± 23.50 | 61.60 ± 27.74 | 51.83 ± 21.51 | 64.50 ± 22.04^“”^ |
|  | SF-36 – Mental health^+^ | 63.69 ± 12.08 | 66.72 ± 18.17 | 64.15 ± 16.26 | 70.24 ± 19.50 |
|  | SF-36 – Vitality score^+^ | 47.12 ± 15.57 | 49.40 ± 22.47 | 51.35 ± 16.94 | 50.60 ± 20.12 |
|  | SF-36 – Mental score^+^ | 54.37 ± 15.82 | 62.10 ± 22.19 | 58.32 ± 21.85 | 62.52 ± 22.43 |
|  | SF-36 – Physical score^+^ | 44.86 ± 16.00 | 57.59 ± 23.32^“”^ | 49.38 ± 20.49 | 57.79 ± 21.07 |

Differential effects between the two subgroups are reported.

^“^ *p* < 0.05 compared with preoperative condition.

^“”^ Significant after FDR correction compared with preoperative condition.

**Legend:** LPD: patients with Parkinson’s disease (PD) who exhibit predominantly left-sided motor symptoms; RPD: patients with PD who exhibit predominantly right-sided motor symptoms; STN DBS: subthalamic nucleus deep-brain stimulation; HC: healthy control; *SD*: standard deviation; SF-36: Short Form 36-item Health Survey.

^+^ The higher the score, the better the quality of life.
